# Human Integrin α_3_β_1_ Regulates TLR2 Recognition of Lipopeptides from Endosomal Compartments

**DOI:** 10.1371/journal.pone.0012871

**Published:** 2010-09-22

**Authors:** Meghan L. Marre, Tanja Petnicki-Ocwieja, Alicia S. DeFrancesco, Courtney T. Darcy, Linden T. Hu

**Affiliations:** 1 Graduate Program in Immunology, Sackler School of Graduate Biomedical Sciences, Tufts University, Boston, Massachusetts, United States of America; 2 Division of Geographic Medicine and Infectious Diseases, Tufts Medical Center, Boston, Massachusetts, United States of America; University Hospital Zurich, Switzerland

## Abstract

**Background:**

Toll-like receptor (TLR)-2/TLR1 heterodimers recognize bacterial lipopeptides and initiate the production of inflammatory mediators. Adaptors and co-receptors that mediate this process, as well as the mechanisms by which these adaptors and co-receptors function, are still being discovered.

**Methodology/Principal Findings:**

Using shRNA, blocking antibodies, and fluorescent microscopy, we show that U937 macrophage responses to the TLR2/1 ligand, Pam_3_CSK_4_, are dependent upon an integrin, α_3_β_1_. The mechanism for integrin α_3_β_1_ involvement in TLR2/1 signaling is through its role in endocytosis of lipopeptides. Using inhibitors of endosomal acidification/maturation and physical tethering of the ligand, we show that the endocytosis of Pam_3_CSK_4_ is necessary for the complete TLR2/1-mediated pro-inflammatory cytokine response. We also show that TLR2/1 signaling from the endosome results in the induction of different inflammatory mediators than TLR2/1 signaling from the plasma membrane.

**Conclusion/Significance:**

Here we identify integrin α_3_β_1_ as a novel regulator for the recognition of bacterial lipopeptides. We demonstrate that induction of a specific subset of cytokines is dependent upon integrin α_3_β_1_-mediated endocytosis of the ligand. In addition, we address an ongoing controversy regarding endosomal recognition of bacterial lipopeptides by demonstrating that TLR2/1 signals from within endosomal compartments as well as the plasma membrane, and that downstream responses may differ depending upon receptor localization. We propose that the regulation of endosomal TLR2/1 signaling by integrin α_3_β_1_ serves as a mechanism for modulating inflammatory responses.

## Introduction

The innate immune response protects the host from microbial invaders through recognition of specific patterns that are recurrent either in pathogens or in the signals they create. The toll-like receptor (TLR) family contains a variety of receptors that recognize a diverse array of these patterns and activate downstream inflammatory cascades [1]. Early models of interactions of TLR signaling proposed simple, direct interactions between TLRs and their ligands, without the aid of other molecules. It is now understood that other adaptor molecules and receptors mediate and alter these interactions resulting in great diversity of responses to different ligands and pathogens recognized by the same receptor [2], [3], [4], [5]. A portion of the diversity is generated by the context and location in which TLRs interact with their ligands [6], [7] and may be further altered by co-stimulation of other pathways that cross talk with a specific TLR [8], [9], [10].

Integrins are divalent, cation-dependent, heterodimeric receptors that mediate a variety of cell-cell and cell-extracellular matrix interactions within host tissues including tissue differentiation, cell migration, and tumor metastases. Roles for integrins in a variety of pathogen recognition and host defense mechanisms are increasingly being recognized. One mechanism by which integrins participate in host defense is by facilitating endocytosis. For example, endocytosis of bacterial pathogens such as enteropathogenic *Yersinia* species [11] and *Staphylococcus aureus* [12], [13] is dependent upon β_1_ integrins. In addition, viruses such as human cytomegalovirus [14] and Kaposi's sarcoma-associated herpes virus [15], [16] are endocytosed via interactions with integrin α_v_β_3_.

Integrins can also participate in host defense through co-operation with other innate immune receptors such as TLRs. Several groups have demonstrated a necessary role for integrin α_M_β_2_ (CD11b/CD18) in the induction of an inflammatory cytokine response to the TLR4 ligand, lipopolysaccharide (LPS) [8], [17], [18]. In addition, a recent publication demonstrated a role for integrin α_v_β_3_ in the regulation of TLR2/1-mediated responses to a number of stimuli including the prototypical bacterial lipopeptide, palmitoyl-3-Cys-Ser-(Lys)_4_ (Pam_3_CSK_4_) [10]. This co-operation was suggested to be mediated through the interaction of Pam_3_CSK_4_ with vitronectin, the extracellular matrix ligand for integrin α_v_β_3_. It was proposed that integrin α_v_β_3_ mediates the attachment of Pam_3_CSK_4_ to macrophages which could lead to clustering of the lipopeptide with the TLR2/1 receptor at the cell surface, thus facilitating signaling.

Integrins also play an important role in the recognition of *B. burgdorferi* [19], [20], [21], [22], an organism that expresses a large number of TLR2 ligands [23], [24], [25], [26]. We have previously shown that *B. burgdorferi* expresses ligands for integrin α_3_β_1_ [27] and that integrin α_3_β_1_ is important for mediating the inflammatory response to *B. burgdorferi* [28]. As a result, we were interested in determining whether integrin α_3_β_1_ may play a role similar to α_v_β_3_ in mediating TLR2 responses to the organism and to purified TLR2 ligands. In this study, we show that human macrophage inflammatory responses to the TLR2/1 ligand Pam_3_CSK_4_ require integrin α_3_β_1_. However, the mechanism by which integrin α_3_β_1_ regulates TLR2/1 function is not through attachment and clustering of ligand at the cell surface as proposed for integrin α_v_β_3_, but rather through the endocytosis of lipopeptides. We further demonstrate that this endocytosis is necessary for the complete response to the lipopeptide. TLR2/1 is classically described as recognizing ligands and activating signaling pathways from the plasma membrane. There remains controversy as to whether TLR2/1 is active within endosomal compartments [29], [30], [31], [32], [33]. In this report, we provide clear evidence using both chemical inhibitors and physical tethering of TLR2/1 ligands that recognition of bacterial lipopeptides, both synthetic and in the context of an intact organism, occurs from within sub-cellular compartments. Recognition of lipopeptides from within endosomal compartments results in the induction of a different subset of inflammatory mediators than recognition from the plasma membrane. Our data provide a new mechanism for the interactions of integrin and TLR receptors and support for the emerging concept that localization and context of TLR-mediated recognition of ligands alters the inflammatory response to a stimulus.

## Results

### Integrin α_3_β_1_ Mediates the U937 Macrophage Response to Pam_3_CSK_4_

**Figure 1:**
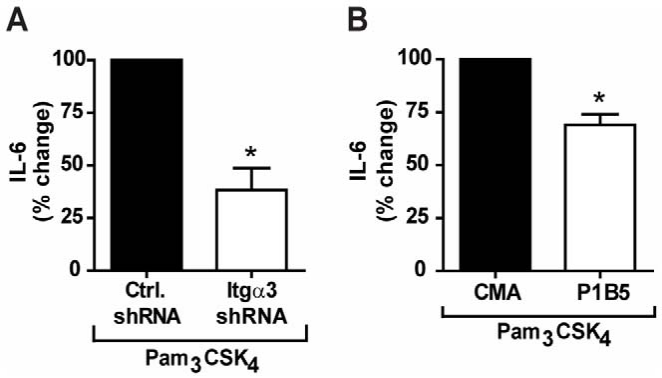
Integrin α_3_β_1_ mediates the U937 macrophage response to Pam_3_CSK_4_. **A**) U937 macrophages stably transduced with integrin α_3_-specific shRNA (Itgα3 shRNA) or non-targeting shRNA (Ctrl. shRNA) were stimulated with 100 ng/ml Pam_3_CSK_4_ for 6 hours under serum-free conditions. Values represent mean secretion of IL-6 relative to control shRNA and S.E.M.of three independent experiments. Cells transduced with control shRNA secreted a mean of 350 pg/ml, and cells transduced with integrin α_3_-targeting shRNA secreted a mean of 61 pg/ml. * p = 0.014 **B**) U937 macrophages were treated with an integrin α_3_β_1_ blocking antibody (P1B5) or a control mouse ascites fluid (CMA) and stimulated with 100 ng/ml Pam_3_CSK_4_ for 6 hours under serum-free conditions. Values represent mean secretion of IL-6 relative to CMA-treated cells and S.E.M.of three independent experiments. CMA-treated cells secreted a mean of 1,068 pg/ml, and P1B5-treated cells secreted a mean of 607 pg/ml. * p = 0.014.

To determine whether integrin α_3_β_1_ cooperates with TLR2/1 signaling, we used shRNA to reduce expression of integrin α_3_ by 73% in U937 macrophage cells (**Fig. S1A**). Specificity of the shRNA construct was confirmed by demonstrating that the shRNA construct did not affect expression of other integrin α chains or TLR2 (**Fig. S1B**). U937 macrophages stably transduced with either non-targeting, control shRNA or integrin α_3_-targeting shRNA were stimulated with the synthetic TLR2/1 ligand Pam_3_CSK_4_ under serum-free conditions. shRNA targeting the integrin α_3_ chain reduced the IL-6 response to Pam_3_CSK_4_ by 62% compared to the control shRNA construct (p = 0.014) (**Fig. 1A**).

To confirm this finding, we tested the effects of antibody blocking of integrin α_3_β_1_ on the response to Pam_3_CSK_4_. Cell cultures were pre-treated with either control mouse ascites fluid (CMA) or an integrin α_3_β_1_ function-inhibiting antibody (P1B5) prior to stimulation with Pam_3_CSK_4_ under serum-free conditions. P1B5 has been demonstrated to specifically inhibit the function of integrin α_3_β_1_, by inhibiting the interaction between integrin α_3_β_1_ and its ligands [34], [35]. Pre-treatment with P1B5 resulted in a 31% decrease in Pam_3_CSK_4_-induced IL-6 secretion compared to pre-treatment with CMA (p = 0.014) (**Fig. 1B**). Taken together, these data suggest that integrin α_3_β_1_ modulates TLR2/1 signaling in response to Pam_3_CSK_4_.

### Exogenous Serum Proteins Do Not Enhance the Role of Integrin α_3_β_1_ in the Inflammatory Response to Pam_3_CSK_4_

**Figure 2:**
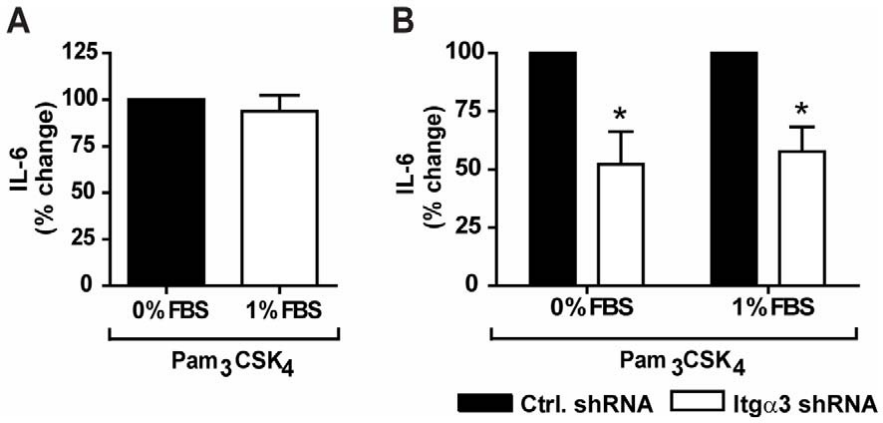
Exogenous serum proteins do not enhance the role of integrin α_3_β_1_. **A**) U937 macrophages were stimulated with 100 ng/ml of Pam_3_CSK_4_ in the presence or absence of 1% FBS for 6 hours. Values represent mean secretion of IL-6 relative to cells stimulated under serum-free conditions and S.E.M. of three independent experiments. Cells stimulated under serum-free conditions secreted a mean of 1,048 pg/ml, and cells stimulated in the presence of 1% FBS secreted a mean of 975 pg/ml. **B**) U937 macrophages stably transduced with integrin α_3_-specific shRNA (Itgα3 shRNA) or non-targeting shRNA (Ctrl. shRNA) were stimulated with 100 ng/ml Pam_3_CSK_4_ in the presence or absence of 1% FBS for 6 hours. Values represent mean secretion of IL-6 relative to control cells and S.E.M. of three independent experiments. Under serum-free conditions, control cells secreted a mean of 1,048 pg/ml and cells transduced with integrin α_3_-targeting shRNA secreted a mean of 545 pg/ml. When stimulated in the presence of 1% FBS, control cells secreted a mean of 974 pg/ml and cells transduced with integrin α_3_-targeting shRNA secreted a mean of 556 pg/ml. * p = 0.037.

Previous work by another group demonstrated that the addition of 1% fetal bovine serum (FBS) to cell culture media dramatically enhanced (8-fold) the pro-inflammatory cytokine response to bacterial lipopeptides. This was shown to function through integrin α_v_β_3_-mediated recognition of vitronectin, its preferred ligand, which binds to bacterial lipopeptides [10]. To determine whether exogenous serum would enhance the role of integrin α_3_β_1_ in facilitating TLR2/1 function, we stimulated U937 macrophages with Pam_3_CSK_4_ in the presence or absence of 1% FBS. Compared to U937 macrophages stimulated under serum-free condition, the addition of 1% FBS did not enhance the secretion of IL-6 (**Fig 2A**). Furthermore, the addition of exogenous serum did not affect the role of integrin α_3_β_1_ in the response to Pam_3_CSK_4_. Indeed, cells transduced with integrin α_3_ shRNA secreted similarly less IL-6 than control cells when stimulated either under serum-free conditions or in the presence of 1% serum (p = 0.037) (**Fig. 2B**). These data suggest that, unlike integrin α_v_β_3_, integrin α_3_β_1_ does not require exogenous serum proteins to regulate the U937 macrophage response to Pam_3_CSK_4_.

### Integrin α_3_β_1_ Does Not Mediate Association of Pam_3_CSK_4_ to U937 Macrophages

**Figure 3:**
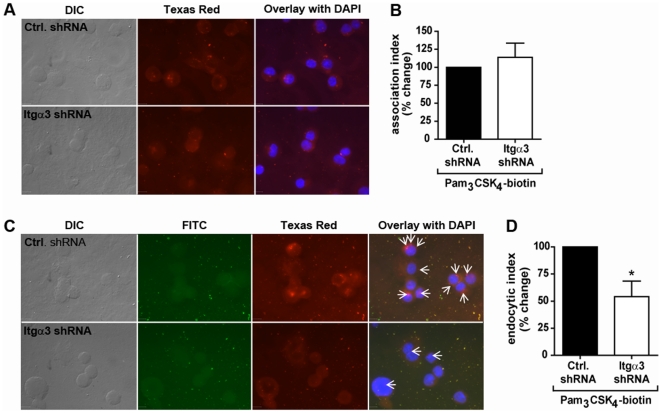
Integrin α_3_β_1_ mediates internalization, but not attachment, of Pam_3_CSK_4_. **A**) U937 macrophages were stably transduced with integrin α_3_-targeting shRNA (Itgα3 shRNA) or non-targeting shRNA (Ctrl. shRNA), stimulated with 5 µg/ml Pam_3_CSK_4_-biotin for 60 minutes, and fixed and stained for immunofluorescent microscopy. Pam_3_CSK_4_-biotin was detected by α-biotin antibodies conjugated to Texas Red. Scale bars, 10 µm. Data are representative of three independent experiments. **B**) The association of Pam_3_CSK_4_-biotin to the macrophages was quantified by determining the association index (the number of cells associated with Pam_3_CSK_4_-biotin divided by total cells). Data represent the mean association index and S.E.M of three independent experiments. The mean association index for control cells was 54.6%, and the mean association index for cells transduced with integrin α_3_-targeting shRNA was 60.6%. **C**) U937 macrophages were stably transduced with integrin α_3_-targeting shRNA (Itgα3 shRNA) or non-targeting shRNA (Ctrl. shRNA) and stimulated with 5 µg/ml Pam_3_CSK_4_-biotin for 60 minutes. The cells were fixed and stained for immunofluorescent microscopy using α-biotin antibodies before (FITC) or after (Texas Red) permeabilization of the cells. Arrows represent internalized Pam_3_CSK_4_-biotin. Scale bars, 10 µm. Data are representative of three independent experiments. **D**) The endocytosis of Pam_3_CSK_4_-biotin was quantified by determining the endocytic index (the number of cells with internalized Pam_3_CSK_4_-biotin divided by number of cells with Pam_3_CSK_4_-biotin associated). Data represent the mean endocytic index and S.E.M. of three independent experiments. The mean endocytic index for control cells was 79.3%, and the mean endocytic index for cells transduced with integrin α_3_-targeting shRNA was 42.9%. * p = 0.037.

The interaction between integrin α_v_β_3_ and vitronectin-lipopeptide complexes was further proposed to mediate macrophage responses by facilitating clustering of TLR2/1 with the lipopeptides at the cell surface [10]. To determine whether integrin α_3_β_1_ affects TLR2/1 responses to Pam_3_CSK_4_ by mediating association of the lipopeptides with macrophages, Pam_3_CSK_4_-biotin was added to U937 cells transduced with control shRNA or integrin α_3_-targeting shRNA. After 60 minutes, the macrophages were fixed, permeabilized, and examined by immunofluorescent microscopy using an anti-biotin antibody conjugated to Texas Red (**Fig. 3A**). The association index was determined by counting the subset of cells with Pam_3_CSK_4_-biotin associated, and expressing this number as a percentage of the total number of cells. No decrease in the association of Pam_3_CSK_4_-biotin to cells transduced with integrin α_3_ targeted shRNA was observed (**Fig. 3A** and **B**). These data suggest that, unlike integrin α_v_β_3_, integrin α_3_β_1_ is not involved in the association of Pam_3_CSK_4_ with macrophages.

### Integrin α_3_β_1_ Mediates Endocytosis of Pam_3_CSK_4_ in U937 Macrophages

Integrins are known to be involved in the internalization of ligands such as extracellular matrix proteins and pathogens or their products [36], [37]. To determine whether integrin α_3_β_1_ participates in endocytosis of Pam_3_CSK_4_, we again employed immunofluorescent methods. In this experiment, U937 macrophages were incubated with Pam_3_CSK_4_-biotin for 60 minutes, then fixed and stained with anti-biotin antibodies before (FITC-labeled) and after (Texas Red-labeled) permeabilization to distinguish Pam_3_CSK_4_-biotin on the surface of cells from that which had been internalized (**Fig. 3C**). The endocytic index was determined by counting the subset of cells to which Pam_3_CSK_4_-biotin molecules attached, and determining the fraction of these cells that had internalized at least one molecule. Knockdown of integrin α_3_ resulted in a 45.9% decrease in internalization of Pam_3_CSK_4_-biotin (p = 0.037) (**Fig. 3D**). These data demonstrate that integrin α_3_β_1_ participates in the endocytosis of Pam_3_CSK_4_.

### Pam_3_CSK_4_ Induces Signaling Through TLR2/1 from Endosomal Compartments and Is Internalized Through Clathrin-Mediated Endocytosis

**Figure 4:**
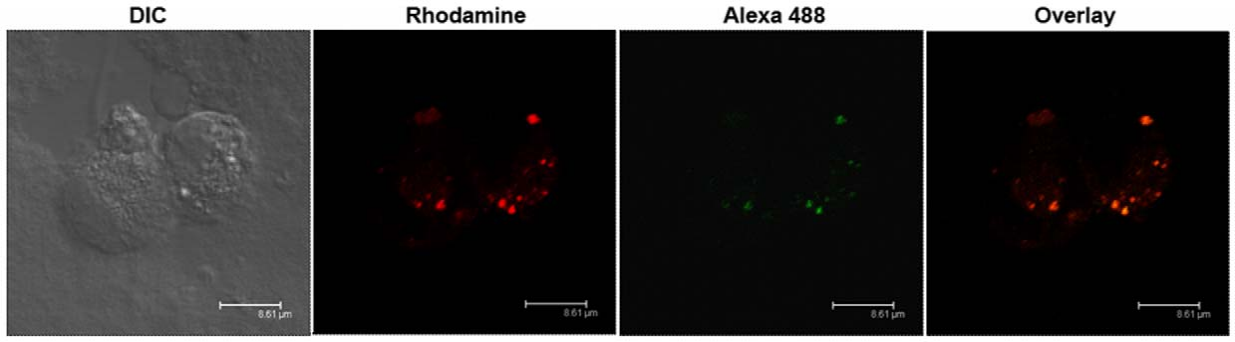
Pam_3_CSK_4_ co-localizes with TLR2 intracellularly. U937 macrophages were stimulated with 5 µg/ml Pam_3_CSK_4_-rhodamine for 20 minutes. The cells were fixed and stained for immunofluorescent microscopy using α-TLR2 antibodies and secondary antibodies conjugated to Alexa Fluor 488. Images show one representative Z stack of 0.7 µm thickness. Scale bars, 8.61 µ µm.

Having shown that integrin α_3_β_1_ mediates uptake of Pam_3_CSK_4_ into sub-cellular compartments, we next sought to determine whether this internalization is important for the inflammatory response to the ligand. To determine whether TLR2 and Pam_3_CSK_4_ are localized together within the cell, we examined co-localization by confocal microscopy. Pam_3_CSK_4_-rhodamine was incubated with U937 macrophages for 20 min and subsequently fixed and stained with anti-TLR2 antibodies, followed by a secondary anti-mouse antibody conjugated to Alexa Fluor 488 (**Fig. 4**). Cells were visualized by confocal microscopy to reveal Pam_3_CSK_4_ and TLR2 intracellular co-localization.

**Figure 5:**
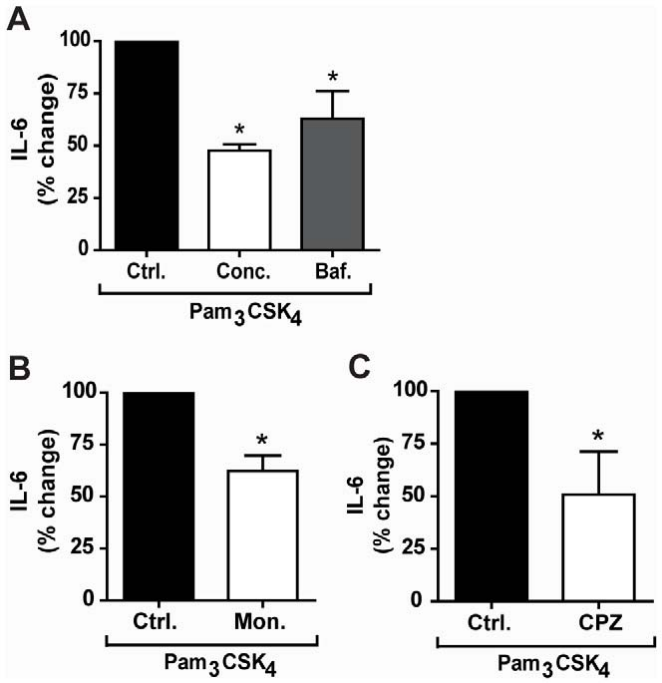
Pam_3_CSK_4_ induces signaling through TLR2/1 from endosomal compartments and is internalized through clathrin-mediated endocytosis. **A**) U937 macrophages were treated with 100 ng/ml concanamycin A (Conc.), 500 µM bafilomycin A1 (Baf.), or control (Ctrl.), and stimulated with 100 ng/ml Pam_3_CSK_4_ for 6 hours under serum-free conditions. Values represent mean secretion of IL-6 relative to control cells and S.E.M. of three independent experiments. Control cells secreted a mean of 670 pg/ml, concanamycin A-treated cells secreted a mean of 320 pg/ml, and bafilomycin A1-treated cells secreted a mean of 430 pg/ml. * p = 0.037 **B**) U937 macrophages were treated with 1 µM monensin (Mon.) or control (Ctrl.), and stimulated with 100 ng/ml Pam_3_CSK_4_ for 6 hours under serum-free conditions. Values represent mean secretion of IL-6 relative to control cells and S.E.M. of three independent experiments. Control cells secreted a mean of 670 pg/ml, and monensin-treated cells secreted a mean of 420 pg/ml, * p = 0.037. **C**) U937 macrophages were treated with 5 µM chlorpromazine (CPZ) or control (Ctrl.) and stimulated with 100 ng/ml Pam_3_CSK_4_ for 6 hours under serum-free conditions. Values represent mean secretion of IL-6 relative to control cells and S.E.M. of three independent experiments. Control cells secreted a mean of 670 pg/ml and CPZ-treated cells secreted a mean of 340 pg/ml. * p = 0.037.

To determine whether intracellular TLR2/1 is able to signal in response to Pam_3_CSK_4_, we pre-treated cells with inhibitors of endosomal acidification and maturation. We first tested the effects of the vacuolar-ATP-ase inhibitors concanamycin A and bafilomycin A1. Pre-treatment of cells with these inhibitors resulted in significant 53% and 37% decreases in IL-6 secretion (p = 0.037) (**Fig. 5A**). To further confirm the importance of endosomal acidification and to rule out a non-specific effect of v-ATPase inhibitors, we also determined the effects of monensin, an antibiotic ionophore, which acts as a Na+/K+ antiporter and inhibits endosomal acidification through a different mechanism. Pre-treatment with monensin also reduced IL-6 secretion by 38% (p = 0.037) (**Fig. 5B**). A caveat to the use of monensin is that it is a known inhibitor of intracellular protein transport. Although we used monensin at concentrations that have not been reported to inhibit protein transport to a significant degree [38], we confirmed our IL-6 ELISA measurements by examining mRNA transcript levels. Quantitative reverse transcriptase PCR (qRT-PCR) analysis of IL-6 transcript confirmed that pre-treatment with monensin reduced this cytokine 32% in Pam_3_CSK_4_ stimulated macrophages (**Fig. S2**). To confirm that these inhibitors do not affect the secretion of IL-6 itself, we pre-treated U937 macrophages with these inhibitors prior to stimulation with TNF-α, which should not require processing in endosomal compartments to induce IL-6. Pre-treatment with either concanamycin A or bafilomycin A1 resulted in no significant change in secretion of IL-6 (data not shown). Pre-treatment with monensin did result in a decrease in IL-6 secretion in response to TNF-α. However, the reduction in IL-6 secretion observed for Pam_3_CSK_4_ stimulation was greater than the decrease observed for TNF-α data not shown). Taken together, these data suggest that endocytosis and endosomal acidification are important for the Pam_3_CSK_4_-induced IL-6 response.

A previous study has suggested that Pam_3_CSK_4_-ovalbumin conjugates are endocytosed by dendritic cells through a clathrin-dependent mechanism. This study did not address whether clathrin-mediated uptake of Pam_3_CSK_4_-ovalbumin (OVA) was through interaction with the lipopeptide or the OVA component [39]. To determine whether endocytosis of Pam_3_CSK_4_ is dependent on clathrin, we tested the addition of chlorpromazine (CPZ), an inhibitor of clathrin-mediated endocytosis [40]. CPZ had a significant effect on the response to Pam_3_CSK_4_, reducing the secretion of IL-6 in U937 macrophages by 49% (p = 0.037) (**Fig. 3C**). These data suggest that endocytosis of Pam_3_CSK_4_ may be clathrin-mediated.

**Figure 6:**
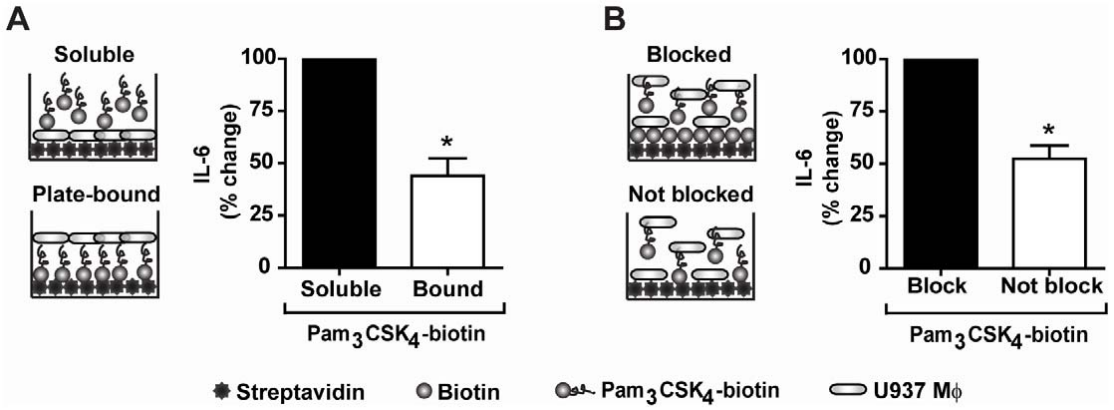
The IL-6 response is dependent upon internalization of Pam_3_CSK_4_. **A**) Schematic of experiment comparing the inflammatory response in cells stimulated with either soluble Pam_3_CSK_4_-biotin or Pam_3_CSK_4_-biotin immobilized on streptavidin plates. U937 macrophages were stimulated with either soluble Pam_3_CSK_4_-biotin or Pam_3_CSK_4_-biotin immobilized on steptavidin plates for 6 hours under serum-free conditions. Values represent mean secretion of IL-6 relative to cells stimulated with soluble Pam_3_CSK_4_-biotin and S.E.M. of three independent experiments. Cells stimulated with soluble Pam_3_CSK_4_-biotin secreted a mean of 1,456 pg/ml, and cells stimulated with plate-bound Pam_3_CSK_4_-biotin secreted a mean of 620 pg/ml. * p = 0.037 **B**) Schematic of experiment comparing the inflammatory response in cells stimulated with Pam_3_CSK_4_-biotin in streptavidin wells either blocked or not with biotin-HRP prior to the addition of cells and Pam_3_CSK_4_-biotin simultaneously. U937 macrophages were stimulated with soluble Pam_3_CSK_4_-biotin in either unblocked streptavidin plates or streptavidin plates blocked with biotin-HRP for 6 hours under serum-free conditions. Values represent mean secretion of IL-6 relative to cells stimulated in blocked wells and S.E.M. of three independent experiments. Cells stimulated in blocked wells secreted a mean of 1,690 pg/ml and cells stimulated in unblocked wells secreted a mean of 895 pg/ml. * p = 0.037.

Because all chemical inhibitors may have off-target effects, we further confirmed the importance of endocytosis of Pam_3_CSK_4_ in the secretion of IL-6 by immobilizing Pam_3_CSK_4_-biotin to streptavidin plates to prevent internalization. Pam_3_CSK_4_-biotin was bound to streptavidin plates overnight and washed prior to the addition of U937 macrophages. As compared to macrophages stimulated with free Pam_3_CSK_4_-biotin, macrophages plated in wells containing plate-bound Pam_3_CSK_4_-biotin secreted 56% less IL-6 (p = 0.037) (**Fig. 6A**). In addition, to ascertain if Pam_3_CSK_4_-biotin “plate-bound” versus “soluble” amounts were comparable, we used a second plate-bound stimulation method. We first blocked the streptavidin wells with biotin-HRP or control. We then added U937 macrophages and Pam_3_CSK_4_-biotin to blocked and unblocked wells simultaneously. In this experiment, a proportion of the Pam_3_CSK_4_ in the control-blocked wells would be expected to bind to streptavidin on the plate, thus reducing the amount of free lipopeptide for endocytosis. We observed a 48% decrease in IL-6 production in the unblocked compared to the blocked wells (p = 0.037) (**Fig. 6B**). This confirms the role of endocytosis of Pam_3_CSK_4_ in inducing TLR2/1-dependent pathways from sub-cellular compartments.

### TLR2/1 Transduces Signals from the Endosome for the Induction of IFN-α1

**Figure 7:**
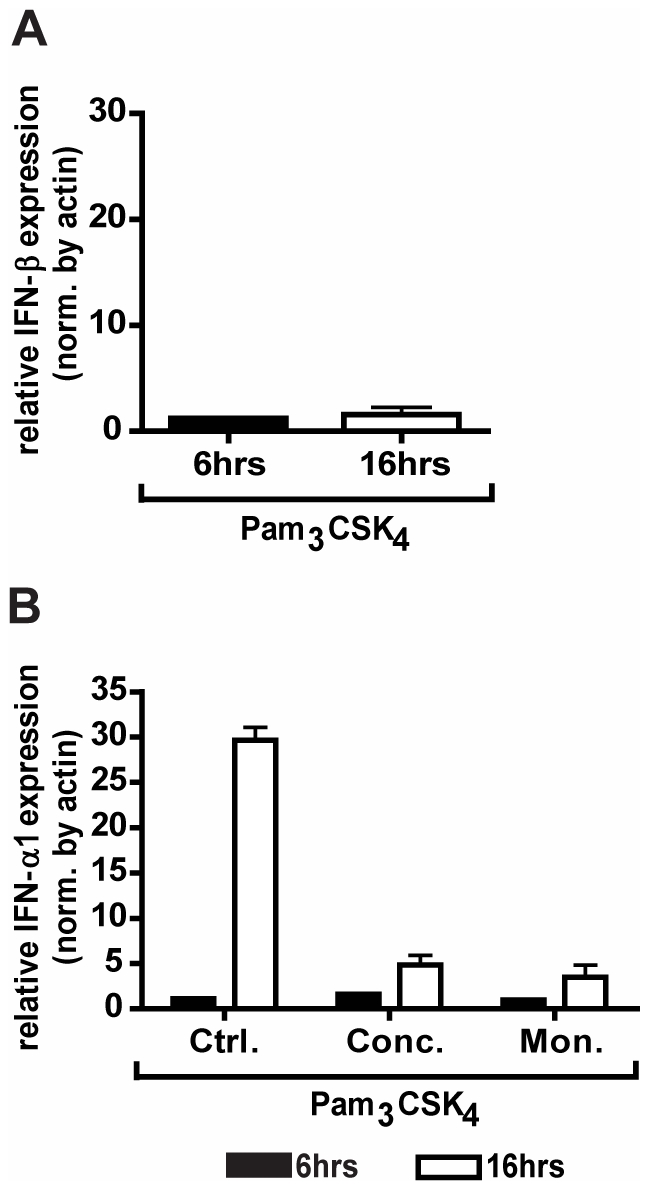
TLR2/1 transduces signals from the endosome for the induction of IFN-α1. **A**) U937 macrophages were treated with 100 ng/ml of Pam_3_CSK_4_ under serum-free conditions for the indicated times. Expression of IFN-β was measured by qRT-PCR. Values represent mean induction of IFN-β expression relative to control cells and S.E.M. of three independent experiments. **B**) U937 macrophages were treated with 100 ng/ml concanamycin A (Conc.), 1 µM monensisn (Mon.), or control (Ctrl.), and stimulated with 100 ng/ml Pam_3_CSK_4_ under serum-free conditions for the indicated times. Expression of IFN-α1 was measured by qRT-PCR. Values represent mean induction of IFN-α1 expression relative to control cells and S.E.M. of three independent experiments. * p = 0.037.

Endosomally located TLR2/1 has been shown to induce type I interferons, specifically IFN-β, in response to viral and bacterial ligands [32], [33]. While multiple studies have shown that TLR2 can activate IFN-β from the endosome [31], [32], we did not observe any induction of IFN-β in U937 macrophage at either 6 hrs or 16 hrs post stimulation (**Fig. 7A**). However, we sought to determine whether sub-cellular localization of TLR2 could induce other type I interferons. We examined the role of Pam_3_CSK_4_ stimulation on induction of IFN-α1, the major IFN-α subtype elicited by human plasmacytoid dendritic cells (pDCs) [41]. U937 macrophages were stimulated with Pam_3_CSK_4_ for 6 and 16 hours in the presence or absence of the endosomal acidification inhibitors concanamycin A and monensin. Induction of IFN-α1 was measured by qRT-PCR. Inhibition of endosomal acidification had a dramatic effect on the transcription of IFN-α1, reducing the transcript levels by 84% for concanamycin-treated cells and 88% for monensin-treated cells (p = 0.037) (**Fig. 7B**). These data demonstrate that Pam_3_CSK_4_ induces an interferon response in U937 macrophages, and that this interferon response requires endocytosis of the ligand.

### TLR2 Mediates the Inflammatory Cytokine Response to *B. burgdorferi* in U937 Macrophages

**Figure 8:**
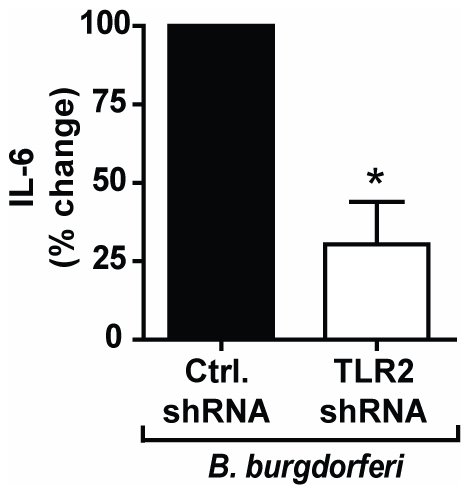
TLR2 mediates the inflammatory cytokine response to *B. burgdorferi* in U937 macrophages. U937 macrophages were stably transduced with TLR2-specific shRNA (TLR2 shRNA) or non-targeting shRNA (Ctrl. shRNA) and stimulated with *B. burgdorferi* MOI 10 for 6 hours under serum-free conditions. Values represent mean secretion of IL-6 relative to control shRNA and S.E.M. of three independent experiments. Cells transduced with control shRNA secreted a mean of 553 pg/ml, and cells transduced with TLR2-targeting shRNA secreted a mean of 142 pg/ml. * p = 0.037.

We have so far demonstrated that integrin α_3_β_1_ mediates the secretion of IL-6 in response to the synthetic TLR2/1 ligand, Pam_3_CSK_4_, by regulating endocytosis of the ligand and facilitating its recognition by TLR2/1 from within endosomal compartments. To confirm the role of integrin α_3_β_1_ and sub-cellular signaling by TLR2/1 in the recognition of lipoproteins presented in the context of a bacterial membrane, we stimulated U937 macrophages with a bacterium that expresses numerous lipoproteins, *B. burgdorferi*. It has previously been reported that TLR2/1 plays the major role in the induction of the inflammatory response to *B. burgdorferi* in macrophages [23], [24], [25], [26]. We first determined the degree to which TLR2 is responsible for the IL-6 response to *B. burgdorferi* in U937 macrophages. Expression of TLR2 mRNA was reduced by 47% in U937 cells by use of an shRNA construct targeting TLR2 mRNA (**Fig. S3A**). Specificity of the shRNA was confirmed by demonstrating that the construct did not affect the expression of other TLRs (**Fig. S3B**). Decreased expression of TLR2 reduced the secretion of IL-6 in response to *B. burgdorferi* by 70% (p = 0.037) (**Fig. 8**). These data suggest that signaling through TLR2 is responsible for the majority of *B. burgdorferi*-induced IL-6 secretion in U937 macrophages.

### Integrin α_3_β_1_ Mediates the Inflammatory Response to *B. burgdorferi* in U937 Macrophages

**Figure 9:**
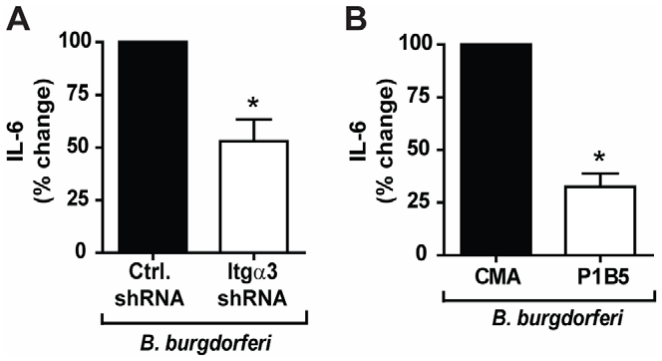
Integrin α_3_β_1_ mediates the inflammatory response to *B. burgdorferi* in U937 macrophages. **A**) U937 macrophages were stably transduced with integrin α_3_-specific shRNA (Itgα3 shRNA) or non-targeting shRNA (Ctrl. shRNA), and stimulated with *B. burgdorferi* MOI 10 for 6 hours under serum-free conditions. Values represent mean secretion of IL-6 relative to control shRNA and S.E.M. of three independent experiments. Cells transduced with control shRNA secreted a mean of 560 pg/ml, and cells transduced with integrin α_3_-targeting shRNA secreted a mean of 310 pg/ml. * p = 0.014 **B**) U937 macrophages were treated with an integrin α_3_β_1_ blocking antibody (P1B5) or control mouse ascites fluid (CMA) and stimulated with *B. burgdorferi* MOI 10 for 6 hours under serum-free conditions. Values represent mean secretion of IL-6 relative to CMA treated cells and S.E.M. of three independent experiments. CMA-treated cells secreted a mean of 1,500 pg/ml, and P1B5-treated cells secreted a mean of 560 pg/ml. * p = 0.014.

It has previously been reported that integrin α_3_β_1_ may play an important role in mediating the inflammatory response to *B. burgdorferi* in human chondrocyte cell cultures [28]. To determine whether integrin α_3_β_1_ regulates the inflammatory response in a macrophage model of infection, we tested the effects of integrin α_3_-targeting shRNA and antibody blocking of integrin α_3_β_1_ on the cellular response to *B. burgdorferi.* shRNA targeting the integrin α_3_ chain reduced the IL-6 response to *B. burgdorferi* by 47% (p = 0.014) (**Fig. 9A**). Pre-treatment with the integrin α_3_β_1_ blocking antibody resulted in a 68% decrease (p = 0.014) in *B. burgdorferi*-induced IL-6 secretion compared to pre-treatment with CMA (**Fig. 9B**), confirming the findings in the shRNA experiments. These data demonstrate that integrin α_3_β_1_ participates in the inflammatory cytokine response to *B. burgdorferi* not only in chondrocytes, but also in macrophages.

### Integrin α_3_β_1_ Mediates Attachment and Endocytosis of *B. burgdorferi* by U937 Macrophages

**Figure 10:**
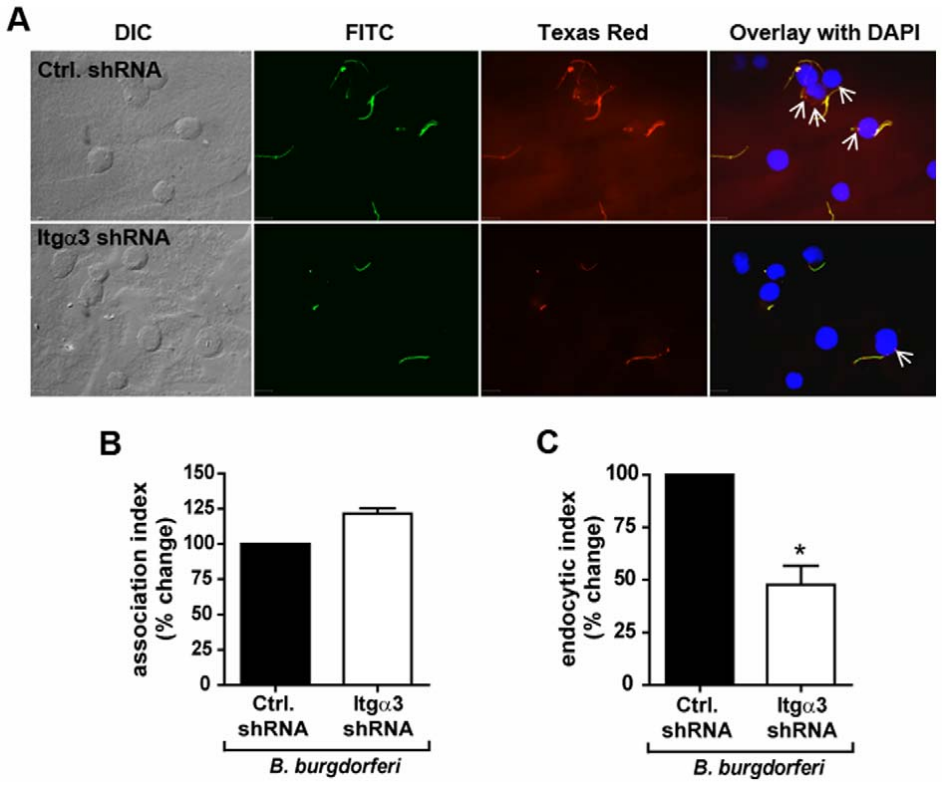
Integrin α_3_β_1_ mediates endocytosis of spirochetes. **A**) U937 macrophages were stably transduced with integrin α_3_-specific shRNA (Itgα3 shRNA) or non-targeting shRNA (Ctrl. shRNA) and stimulated with *B. burgdorferi* MOI 10 for 60 minutes under serum-free conditions. Endocytosis of spirochetes was determined by immunofluorescent staining before (FITC-labeled) and after (Texas Red-labeled) permeabilization of the cells. Arrows indicate internalized spirochetes. Scale bars, 10 µm. Data are representative of three independent experiments. **B**) The association of *B. burgdorferi* with the macrophages was quantified by determining the association index (the number of cells associated with *B. burgdorferi* divided by the total number of cells). Data represent the mean association index and S.E.M. of three independent experiments. The mean association index for control cells was 51.1%, and the mean association index for cells transduced with integrin α_3_-targeting shRNA was 62.2%. **C**) The endocytosis of *B. burgdorferi* was quantified by determining the endocytic index (the number of cells with *B. burgdorferi* internalized divided by the number of cells with *B. burgdorferi* associated). Data represent the mean endocytic index and S.E.M. of three independent experiments. The mean endocytic index for control cells was 41.8%, and the mean endocytic index for cells transduced with integrin α_3_-targeting shRNA was 20.1%. * p = 0.037.

To determine whether integrin α_3_β_1_ regulates the inflammatory response to *B. burgdorferi* by regulating association with the macrophages and subsequent endocytosis, U937 macrophages were stably transduced with shRNA targeting integrin α_3_ or control prior to stimulation with the spirochetes. At 60 minutes, the macrophages were fixed and visualized by immunofluorescent microscopy using an anti-*B. burgdorferi* polyclonal antibody and fluorescently labeled secondary antibodies. Integrin α_3_-targeting shRNA did not reduce the association index (**Fig. 10A** and **B**). However, integrin α_3_ shRNA did inhibit endocytosis of the organism, decreasing the endocytic index by 53% (p = 0.037) (**Fig. 10A** and **C**). These results suggest that, like its role in the response to Pam_3_CSK_4_, integrin α_3_β_1_ regulates the endocytosis, but not the association, of *B. burgdorferi* in U937 macrophages.

### Induction of Inflammatory Cytokines by *B. burgdorferi* Occurs Downstream of Endocytosis and Endolysosomal Processing

**Figure 11:**
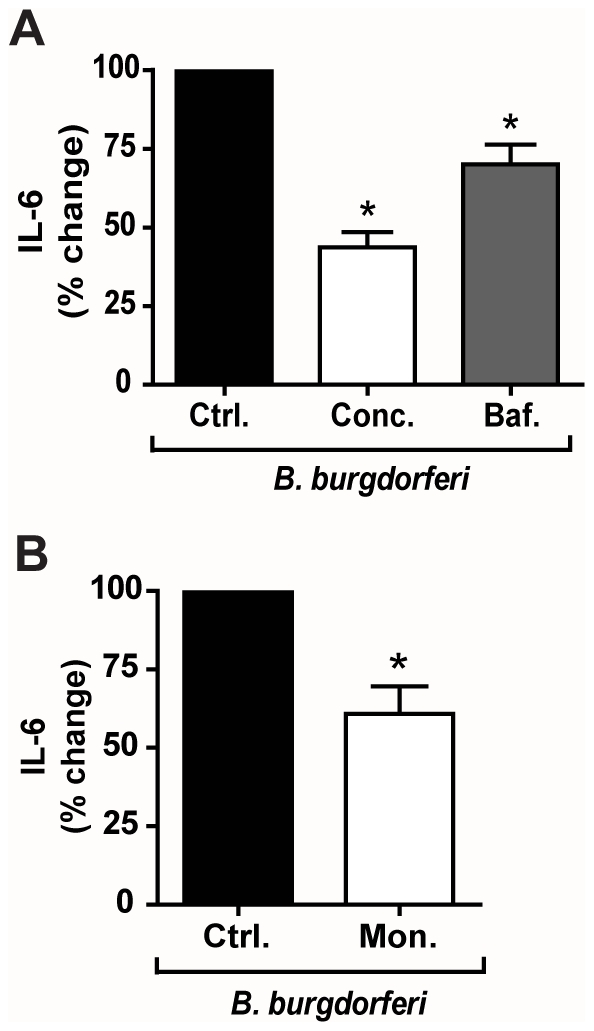
Induction of IL-6 by *B. burgdorferi* occurs downstream of endocytosis and endolysosomal processing of spirochetes. **A**) U937 macrophages were treated with 100 ng/ml concanamycin A (Conc.), 500 µM bafilomycin A1 (Baf.), or control (Ctrl.) and stimulated with *B. burgdorferi* MOI 10 for 6 hours under serum-free conditions. Values represent mean secretion of IL-6 relative to control cells and S.E.M. of three independent experiments. Control cells secreted a mean of 955 pg/ml, concanamycin A-treated cells secreted a mean of 437 pg/ml, and bafilomycin A1-treated cells secreted a mean of 680 pg/ml. * p = 0.037 **B**) U937 macrophages were treated with 1 µM monensin (Mon.) or control (Ctrl.), and stimulated with *B. burgdorferi* MOI 10 for 6 hours under serum-free conditions. Values represent mean secretion of IL-6 relative to control cells and S.E.M. of three independent experiments. Control cells secreted a mean of 955 pg/ml and monensin-treated cells secreted a mean of 567 pg/ml. * p = 0.037.

To determine whether acidification and endosomal maturation is important in inflammatory signaling in response to *B. burgdorferi*, we tested inhibitors that were used for the above studies with Pam_3_CSK_4_. The addition of either concanamycin A, bafilomycin A1 or monensin to U937 cells prior to the addition of *B. burgdorferi* inhibited induction of IL-6 by 56%, 30% and 40% respectively (p = 0.037) (**Fig. 11A** and **B**). Monensin ELISA data was again confirmed by qRT-PCR. The IL-6 transcript was reduced 51% upon monensin pre-treatment of *B. burgdorferi*-stimulated macrophages (**Fig. S4**). These studies with inhibitors of endosomal acidification support the concept that endosomal maturation and bacterial digestion within the endosome are important in eliciting a full pro-inflammatory host response to *B. burgdorferi*.

## Discussion

The inflammatory response of macrophages to bacteria involves the engagement of many different receptors both on the cell surface and in sub-cellular compartments. The mechanisms by which different receptors interact to mediate inflammation are only beginning to be understood. Here, we have demonstrated that integrin α_3_β_1_ co-operates with TLR2/1 to facilitate inflammatory responses to bacterial lipopeptides by macrophages. Inhibition or knockdown of integrin α_3_β_1_ inhibits inflammatory responses by macrophages to both the prototypic TLR2/1 ligand, Pam_3_CSK_4_, and to live *B. burgdorferi*, an organism that expresses numerous TLR2/1 lipoprotein ligands. The mechanism we have identified is through the role of integrin α_3_β_1_ in mediating the endocytosis of Pam_3_CSK_4_ and *B. burgdorferi*, thus facilitating the recognition of ligands by TLR2/1 within the endosome. Using shRNA, blocking antibodies, and fluorescent imaging, we have clearly demonstrated that Pam_3_CSK_4_ is endocytosed by macrophages in an integrin α_3_β_1_-dependent manner. Using acidification inhibitors and tethering of lipopeptides, we have shown that endocytosis is necessary for induction of IL-6 and IFN-α1.

Although another integrin, α_v_β_3_, has also been reported to regulate TLR2/1-mediated macrophage responses [10], our data shows that integrin α_3_β_1_ mediates TLR2/1 interactions with its ligands through a different mechanism. Integrin α_v_β_3_ was proposed to mediate attachment of Pam_3_CSK_4_ to cell surfaces through binding of vitronectin attached to the lipopeptide. Our results differ in that the addition of exogenous serum (which contains both vitronectin and ligands for integrin α_3_β_1_) does not affect the inflammatory response to Pam_3_CSK_4_ in U937 macrophages. In addition, since the majority of our experiments were performed in serum-free media, we have shown that the absence of exogenous serum does not affect the requirement for integrin α_3_β_1_ in facilitating Pam_3_CSK_4_ induction of IL-6. The fact that down-regulation of integrin α_3_ does not decrease attachment of Pam_3_CSK_4_ to the macrophages further supports the case that integrin α_3_β_1_ plays a different role than integrin α_v_β_3_ in facilitating TLR2/1 signaling.

Although integrins are being increasingly recognized to be important mediators of internalization of host factors as well as bacterial and viral ligands and pathogens [36], this is the first report demonstrating that an integrin mediates the endocytosis of synthetic bacterial lipopeptides. Integrin-associated mechanisms of endocytosis include recruitment of clathrin, caveloin, and dynamin to the endocytic cup [42] which is consistent with our observations that chemical inhibition of clathrin also blocks IL-6 induction in response to Pam_3_CSK_4_.

Whether integrin α_3_β_1_ plays a similar role in facilitating responses to live bacterial pathogens was determined by testing responses to *B. burgdorferi*, a bacterium characterized by its high concentration of lipoproteins. We demonstrated that integrin α_3_β_1_ is important in the endocytosis of this organism, facilitating the recognition of borrelial ligands within endolysosomal compartments by receptors including TLR2/1. Although we cannot rule out the involvement of other endosomal receptors (primarily TLR7 [43]; *B. burgdorferi* does not activate TLR4 [44] and U937 macrophages are unresponsive to TLR9 ligands [45]), our data show that TLR2/1 plays the major role in regulating the IL-6 response to *B. burgdorferi*. We have previously published that integrin α_3_β_1_ mediates TLR2-independent signaling in human chondrocytes in response to *B. burgdorferi* stimulation [28]. There are several possible explanations that would be consistent with our current findings. First, the function of integrin α_3_β_1_ may be different between cell types. Second, integrin α_3_β_1_ may induce some direct signaling for the induction of inflammatory cytokines, but the contribution of integrin α_3_β_1_ signaling is minor in comparison to its TLR2-mediated effects. Finally, the primary contribution of integrin α_3_β_1_ may be the endocytosis of *B. burgdorferi* or its ligands. This endocytosis may still occur in the absence of TLR2, with activation of other endosomal TLRs leading to the induction of inflammatory cytokines.

**Figure 12:**
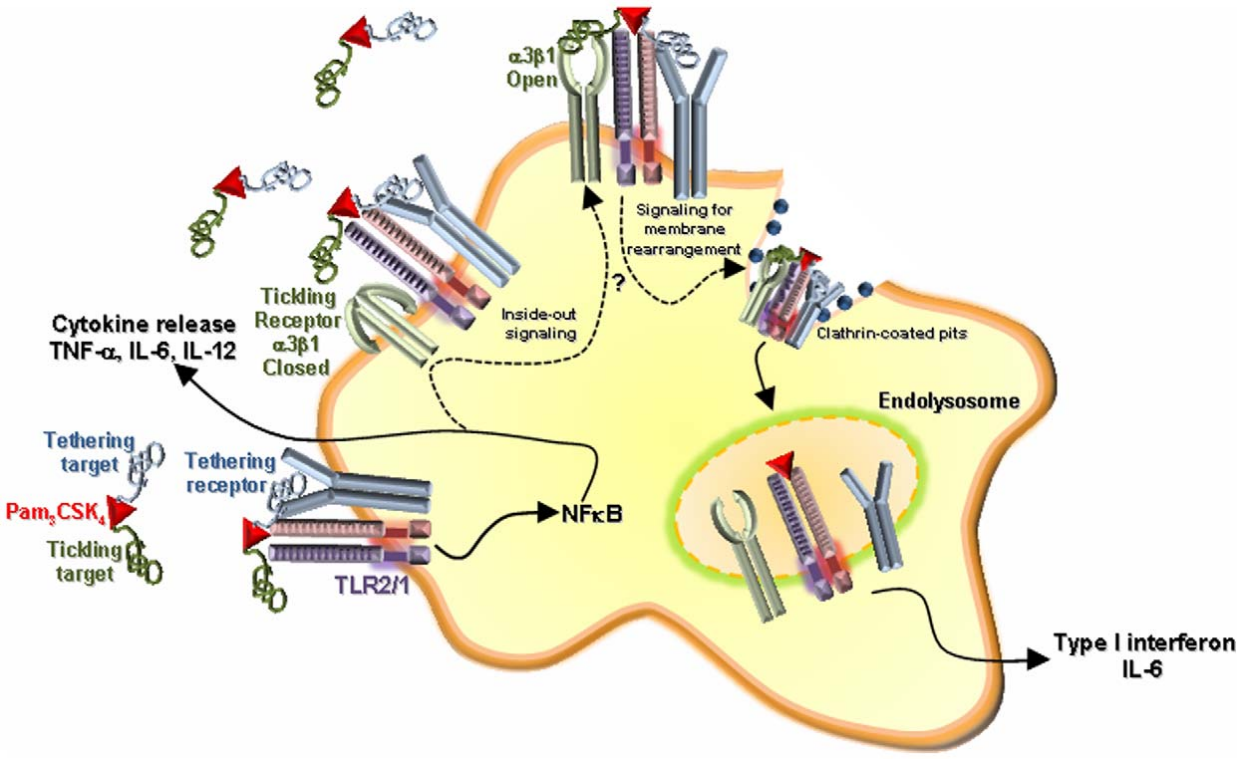
Model. Free bacterial lipopeptide is bound to host proteins which act as ligands for a “tethering receptor”, anchoring Pam_3_CSK_4_ to the cell and bringing it into proximity with TLR2 to initiate TLR2-mediated signaling at the cell surface. In addition to the induction of pro-inflammatory cytokines, the activation of cells via TLR2 may contribute to inside-out signaling, causing a shift in integrin α_3_β_1_ conformational equilibrium from an inactive (“closed”) to an active (“open”) conformation. Once active, integrin α_3_β_1_ serves as a tickling receptor, participating in the endocytosis of Pam_3_CSK_4_ through clathrin-mediated mechanisms. Upon internalization, Pam_3_CSK_4_ and TLR2 co-localize in the endosome. TLR2 signals from this endosomal compartment for the induction of a second subset pro-inflammatory cytokines such as IL-6 and type I interferons.

Our current model for the role of integrin α_3_β_1_ in facilitating TLR2 signaling is shown in figure 12. Because binding of either Pam_3_CSK_4_ or *B. burgdorferi* to cells is independent of integrin α_3_β_1_, we propose that lipoproteins attach to the cell through a “tethering receptor”. This attachment brings the ligand into proximity with TLR2/1. We further propose that clustering of Pam_3_CSK_4_, the tethering receptor, and TLR2 initiates inside-out signaling to activate integrin α_3_β_1_, which acts as a “tickling receptor” to facilitate endocytosis of the receptor complex. There is precedent that internalization of particles or ligands can involve a series of receptors that separately mediate attachment and internalization [46], [47], [48]. Once localized within the endosome, TLR2/1 recruits adaptor molecules such as MyD88 that then activate pathways responsible for induction of inflammatory cytokines.

There has been significant controversy regarding whether TLR2/1 is active within endosomal compartments. TLR2 is clearly recruited to endosomal membranes [49], but its ability to signal from these compartments has been questioned. It has been suggested that TLR2 can only signal from the plasma membrane because its adaptor, TIRAP, does not localize to intracellular compartments [31]. In this model, the TIRAP/MyD88 adaptor complex dissociates from TLR2 prior to its inclusion in an endolysosomal membrane, leaving it unable to signal. However, other studies have shown that signaling defects caused by TIRAP deficiency can be overcome by higher levels of Pam_3_CSK_4_ stimulation [50] and TIRAP deficient mice are still capable of an inflammatory response to TLR2 ligands [30], [51] suggesting that the lack of recruitment of TIRAP to endolysosomes does not exclude the possibility of TLR2 signaling from these compartments.

Recently, Barbalat et al. showed that TLR2 signals from endosomal compartments of specialized mouse inflammatory monocytes in response to virus, but not in response to Pam_3_CSK_4_ [32]. However, a subsequent study by Dietrich et al., which was published while this manuscript was under review at another journal, showed that synthetic bacterial lipopeptides can also signal from endosomal compartments of murine bone marrow-derived macrophages [33]. Our data, using similar, as well as different, techniques than in the above reports, also shows that bacterial lipopeptides are recognized from within endosomes by TLR2/1, providing strong evidence to resolve this issue.

While our study and the studies by Barbalat et al. and Dietrich et al. agree that TLR2/1 signals from the endosome, there were differences seen in the character of the inflammatory response generated. One study showed that TLR2 signals for IFN-α and IFN-β induction in response to virus, but not in response to Pam_3_CSK_4_ [32]. In contrast, another study showed that bacterial TLR2 ligands stimulate the induction of IFN-β [33]. We did not observe induction of IFN-β in response to Pam_3_CSK_4_ in human U937 macrophages; however, this may be due to differences in cell type since we did confirm Pam_3_CSK_4_-induced IFN-β in murine bone marrow derived macrophages (data not shown). We also found that bacterial lipopeptide stimulation can indeed result in the induction of IFN-α. We found that both *B. burgdorferi* (data not shown) and Pam_3_CSK_4_ induce mRNA for IFN-α1 in U937 macrophages and that this induction could be almost completely inhibited by the addition of endosomal acidification inhibitors. The discrepancy between our data and previous reports which showed no effect of Pam_3_CSK_4_ on IFN-α induction is likely due to the examination of different subtypes of IFN-α in different cell types [32], [33].

Our study also differs from previous claims that TLR2/1 does not induce pro-inflammatory cytokines from the endosome [31], [32], [33] as we show clear evidence that internalization and endosomal acidification is necessary for an IL-6 response. It has been suggested that the involvement of the IFN-β autocrine/paracrine loop enhances NF-κB-mediated induction of IL-6. It is unclear how much IFN-β can contribute to the enhancement of IL-6 production, as studies addressing this point in different cell types and downstream of different stimuli have produced variable results [52], [53], [54], [55], [56]. In U937 macrophages, our data show no induction of IFN-β in response to Pam_3_CSK_4_, suggesting that IL-6 secretion in our system is not controlled by IFN-β. Therefore, the decrease we observe in IL-6 production upon treatment with endosomal acidification inhibitors is likely due to the more classical endosomal TLR-mediated induction of IL-6 through NF-κB.

Localization of TLR2 has been suggested to generate specificity in the inflammatory response [32], [33]. Although induction of IL-6 by Pam_3_CSK_4_ was significantly decreased by endosomal acidification inhibitors, the same inhibitors had much less effect on TNF-α production by U937 macrophages (**Fig. S5**). This is consistent with the observations in murine macrophages [33]. Conversely, we found that the effects of endosomal acidification inhibitors on IFN-α1 were more pronounced than the effects on IL-6, suggesting a greater dependence on endosomal signaling for IFN-α1. The fact that different cytokines induced by Pam_3_CSK_4_ are affected differentially by acidification inhibitors suggests that TLR2/1 responses are likely to be context dependent, in that, signaling from TLR2/1 localized to the plasma membrane may differ from signaling activated by TLR2/1 in endosomes. One could hypothesize that induction of TNF-α occurs primarily from plasma membrane-localized TLR2/1, that induction of IFN-α1 and IFN-β occurs primarily from endosomally localized TLR2/1, and that IL-6 may be induced by both plasma and endosomally localized TLR2/1. The differences in cytokine profiles resulting from context-dependent TLR2 signaling, as well as the mechanisms by which cellular context alters TLR2/1 signaling, remain to be determined.

The importance of integrin α_3_β_1_ in the recognition of bacterial lipopeptides and host defense in an *in vivo* model is unknown. Mice with integrin α_3_ deficiency die early after birth and, to our knowledge, there are no cohorts of human subjects deficient in integrin α_3_β_1_. Patients with leukocyte adhesion deficiency type III (LAD III) harbor a mutation in the KINDLIN3 gene which inhibits the activation of members of the β_1_, β_2_, and β_3_ integrin families [57]. These patients are highly susceptible to multiple different infections. Whether the increased susceptibility to infection is caused by loss of integrin α_3_β_1_ function specifically will require further research.

In the model presented in Fig. 12, there are still aspects that will require further investigation. The identity of the tethering receptor that binds Pam_3_CSK_4_ to the surface of macrophages has not been identified. It is tempting to speculate that integrin α_v_β_3_ is responsible for attachment of Pam_3_CSK_4_ to the surface of the macrophages, since integrin α_v_β_3_ was previously proposed to mediate attachment of Pam_3_CSK_4_ to cell surfaces [10]. Another candidate molecule that could serve as a tethering receptor is the TLR2 and TLR4 adaptor molecule CD14. CD14 has been shown to serve as a tethering receptor in other systems [48] and is known to interact with TLR2/1 in the recognition of Pam_3_CSK_4_ [4], [5]. Further work will be necessary to determine which receptors serve as tethering receptors to facilitate integrin α_3_β_1_-mediated endocytosis of Pam_3_CSK_4_.

Our model also includes the possibility that signaling from TLR2/1 activated on the plasma membrane results in “inside-out” signaling for the activation of integrin α_3_β_1_. Integrins exist in the plasma membrane in a state of equilibrium between active and inactive conformations. The balance between these two states can be shifted toward the open conformation by signaling pathways, which are initiated by the ligation of other cellular receptors and ultimately lead to the activation of the integrin [36]. Prior work in our laboratory has shown that MyD88 activation is important in mediating endocytosis of *B. burgdorferi* through activation of phosphoinositide 3-kinase (PI3-K) [24], [58]. Activation of PI3-K can result in membrane conformational changes that activate integrins [59]. Further work is required to determine whether TLR2-mediated signals are important for the activation of integrin α_3_β_1_.

In summary, we have demonstrated three important findings in this study. First, induction of IL-6 in response to bacterial lipopeptides is mediated through integrin α_3_β_1_. Second, the complete pro-inflammatory cytokine response to both Pam_3_CSK_4_ and *B. burgdorferi* requires α_3_β_1_ integrin-mediated endocytosis and subsequent maturation of the endolysosomes, demonstrating that TLR2/1-mediated induction of pro-inflammatory cytokines and IFN-α1 occur from sub-cellular compartments. And finally, signaling through TLR2/1 may be context-dependent with activation of different downstream pathways from the plasma membrane and from endolysosomal compartments. We therefore propose a model in which integrin α_3_β_1_ mediates the endocytosis of bacterial lipopeptides, thus facilitating the recognition of these ligands and subsequent initiation of signaling cascades by endosomal TLR2/1.

## Materials and Methods

### Cell Cultures and Reagents

The human monocyte cell line U937 (American Type Culture Collection) was maintained in RPMI (Mediatech) with 10% FBS and 1% penicillin-streptomycin. For all experiments, U937 monocytes were differentiated at a concentration of 5×10^5^ cells per well in 24-well plates with 100 nM phorbol 12-myristate 13-acetate (Sigma) for 48 hours. All experiments were performed under serum-free conditions, unless otherwise noted.

The TLR2/1 triacylated lipid ligand, Pam_3_CSK_4_ (Invivogen), biotinylated Pam_3_CSK_4_ (Axxora) or Pam_3_CSK_4_-rhodamine (Invivogen) were resuspended in endotoxin-free water. Pam_3_CSK_4_ was used at a concentration of 100 ng/ml to stimulate macrophages, and Pam_3_CSK_4_-biotin and Pam_3_CSK_4_-rhodamine were used at indicated concentrations.

Clonal isolates of infectious, low passage *B. burgdorferi* sensu stricto (strain N40, clone D10E9) were cultured in Barbour-Stoenner-Kelley (BSK II) medium at 37°C as described [60], and used at multiplicity of infection (MOI) 10∶1.

### shRNA

Lentiviral plasmid vectors (pLKO.1) encoding non-targeting shRNA, integrin α_3_-targeting shRNA, or TLR2-targeting shRNA (Sigma-Aldrich) were packaged into lentiviruses following the manufacturer's instructions. Briefly, HEK293 cells were transfected with shRNA vectors and packaging vectors (Sigma-Aldrich) using FuGENE6 (Roche). Supernatants were harvested 48 and 72 hours post-transfection and stored at −80°C. To reduce expression of target genes in U937 cells, the monocytes were incubated with control, integrin α_3_-targeting, or TLR2-targeting virus for 20 hours at 37°C. The media was then replaced with fresh RPMI for 24 hours. Positively transduced cells were selected with 6 µg/ml of puromycin for 24 hours. These cells were then differentiated as described above. After differentiation, the cells were harvested in TRIzol, and mRNA was isolated as described below. From this mRNA, cDNA was synthesized and qRT-PCR analysis was performed to determine the relative expression of integrins and TLRs. Of the five different gene-specific shRNA constructs tested, the data presented in this paper were obtained with the construct which best reduced expression of the target mRNA with no impact on other integrins or TLRs. The shRNA construct targeting integrin α_3_ was 5′ - CCTCTATATTGGGTACACGAT -3′. The shRNA construct targeting TLR2 was 5′-CCCATGTTACTAGTATTGAAA -3′. In each experiment performed, some cells were examined by qRT-PCR to confirm the reduction of the expression of target genes.

### Inhibitors and Blocking Antibodies

Inhibition of endosomal acidification was achieved using inhibitors of V-ATPase, concanamycin A (100 ng/ml) and bafilomycin A1 (500 µM) (Sigma) or the ionophore monensin (1 µM) (Sigma). Clathrin-mediated endocytosis was inhibited with chlorpromazine (CPZ) (5 µM) (Sigma). Concentrations were chosen based on prior studies [40], [61]. All inhibitors were added 30 minutes prior to stimulation. For CPZ experiments, the media was replaced at the time of stimulation to remove CPZ.

For blocking antibody experiments, U937 macrophages were incubated for 2 hours prior to stimulation with 50 µg/ml of control mouse ascites fluid (NS-1 murine myeloma, Sigma-Aldrich) or anti-integrin α_3_β_1_ monoclonal antibody (P1B5, Millipore).

### ELISA

Supernatants were collected 6 hours post stimulation. IL-6 and TNF-α were measured using the DuoSet enzyme linked immunoabsorbent assay (ELISA) kit (R&D systems) following the manufacturer's instructions.

### Ligand Tethering Experiments

High sensitivity streptavidin-coated plates (Pierce, ThermoScientific) were coated with 10 µg/ml of Pam_3_CSK_4_-biotin in PBS or control and washed prior to the addition of U937 macrophages. In control wells, soluble Pam_3_CSK_4_-biotin was added after allowing U937s cells to settle. In experiments with blocked wells, wells were coated with 20 µg/ml biotin-HRP (Invitrogen) in PBS or control and washed prior to the simultaneous addition of U937 cells and 10 µg/ml Pam_3_CSK_4_-biotin.

### Endocytosis Assay

For Pam_3_CSK_4_ endocytosis experiments, U937 monocytes stably transduced with integrin α_3_-targeting shRNA or control shRNA were differentiated in wells containing glass coverslips. After differentiation, 5 ug/ml of Pam_3_CSK_4_-biotin or Pam_3_CSK_4_-rhodamine were added. After 60 minutes at 37°C for Pam_3_CSK_4_-biotin or 20 min at 37°C for Pam_3_CSK_4_-rhodamine, the cells were washed three times in cold PBS, fixed in 3.7% paraformaldehyde, and stained for immunofluorescent microscopy.

*B. burgdorferi* endocytosis experiments were performed according to a similar protocol. U937 monocytes stably transduced with integrin α_3_-targeting shRNA or control shRNA were differentiated in wells containing glass coverslips. After the addition of *B. burgdorferi*, the plates were centrifuged at 300× g at 4°C for 5 minutes. After 60 minutes at 37°C, the cells were washed three times in cold PBS, fixed in 3.7% paraformaldehyde, and stained for immunofluorescent microscopy.

### Microscopy

Immunofluorescent microscopy was performed as previously described [24], [58] with modifications. For Pam_3_CSK_4_ endocytosis experiments, cells on coverslips were incubated with an anti-biotin FITC-conjugated polyclonal goat antibody (Novus Biologicals) at a 1∶500 dilution to label extracellular Pam_3_CSK_4_-biotin. Cells were then washed three times for 5 minutes in PBS and permeabilized with −20°C methanol. Cells were then incubated with anti-biotin Texas Red-conjugated polyclonal goat antibody (Novus Biologicals) at a 1∶500 dilution to label both extracellular and intracellular Pam_3_CSK_4_-biotin. The coverslips were mounted using 4′,6-diamidino-2-phenylindole in Vectashield mounting medium (Vector Laboratories).

For *B. burgdorferi* endocytosis experiments, cells on coverslips were incubated with an anti-*B. burgdorferi* polyclonal rabbit antibody (gift from Dr. Allen Steere) at a 1∶10,000 dilution, then washed and incubated with a FITC-conjugated goat anti-rabbit IgG antibody (Molecular Probes) to stain extracellular bacteria. Cells were washed three times for 5 minutes in PBS and permeabilized with −20°C methanol. Cells were again incubated with anti-*B. burgdorferi* antibody, followed by a Texas Red-conjugated goat anti-rabbit IgG antibody (Molecular Probes) to stain both extracellular and intracellular bacteria. Coverslips were mounted in Vectashield mounting medium.

Coverslips were examined using a Zeiss Axiolan 2 microscope. Images were captured with a digital CCD camera (Hamamatsu). Images were merged using Volocity software (Improvision Inc.). The association index was determined by dividing the number of macrophages with at least one Pam_3_CSK_4_-biotin molecule or bacterium associated (either external or internal) by the total number of macrophages. The endocytic index was determined by dividing the number of cells that had internalized at least one molecule or bacteria by the number of cells associated with molecules or bacteria.

For confocal microscopy studies, cells were permeabilized and incubated with an anti-TLR2 antibody (clone TLR2.1, Invivogen) at 1∶50 dilution, then washed and incubated with an anti-mouse Alexa Fluor 488-conjugated secondary antibody to detect intracellular and extracellular TLR2.

Confocal microscopy was performed at the Tufts Imaging Core Facility using the Leica TCS SP2 AOBS microscope using the Argon 488 nm and HeNe 568 nm red diode lasers. For simultaneous green and red channel imaging, the multitracking function was utilized and each laser was activated one at a time, ensuring no cross-talk occurred between the two fluorochromes. Z stack images of 0.7 µm were captured using the 63X oil objective and analyzed using the Leica Confocal Software (Leica).

### Quantitative PCR

Cells were collected at 6 hours post infection unless otherwise indicated. RNA was extracted using TRIzol (Invitrogen) following the manufacturer's instructions. RNA was resuspended in water containing RNaseOut (Invitrogen) and treated with DNaseI using the Turbo DNA-free kit (Ambion). cDNA was synthesized using the ImPromII kit (Promega) following the manufacturer's instructions. Quantification of cDNA was performed by quantitative RT-PCR (iCycler, BioRad) using the iQ SYBR Green Supermix (BioRad). Cycling parameters were 95°C for 15 minutes followed by 40 cycles of 95°C for 30 seconds, 60°C for 30 seconds, and 72°C for 30 seconds. Primers used to measure IL-6, TNF-α, and β-actin were published previously [28], [62]. Human integrin α_3_ primers, forward: 5′-CCCGCTATTATCAGATCATGCC-3′, reverse 5′-CAGTAGTATTGGTCCCGAGTCT-3′ were generated by Primer Bank, ID# 6006011a1. Human integrin α_v_ primers, forward: 5′-AATGTGACTGGTCTTCTACCCG-3′, reverse 5′-ACCACTGATGGGACTTAAATTCC-3′ were generated by Primer Bank, ID# 4504763a2. Human integrin α_5_ primers, forward: 5′-TTCTGGAGTATGCACCCTGC-3′, reverse 5′-TGGTCCACCTAAAACCACACG-3′ were generated by Primer Bank, ID# 4504751a3. Human integrin α_6_ primers, forward: 5′-TCGGCACAGCAACCTTGAA-3′, reverse 5′-TTGTGAGACTCCTTTTCCAATC-3′ were generated by Primer Bank, ID# 30046796a2. Human TLR2 primers, forward: 5′-CCAGCACACGAATACACAGT-3′, reverse 5′- CAAATGAAGTTATTGCCACC-3′. Human TLR4 primers, forward: 5′-TACAAAATCCCCGACAACCTCC-3′, reverse 5′-GCTGCCTAAATGCCTCAGGG-3′ were generated by Primer Bank, ID# 19924149a1. Human TLR7 primers, forward: 5′-GGAACGGGTACCAAAATGGTGTTTCCAATGTGG-3′, reverse 5′-TAATCTGGATCCGACCGTTTCCTTGAACACCTG-3′. Human TLR9 primers, forward: 5′-GCGACCAGGCTCCCGAAGG-3′, reverse 5′-GTGTCCTTTGCCCACCTGTCTC-3′. Human IFN-α1 primers, forward: 5′-GCCTCGCCCTTTGCTTTACT-3′, reverse: 5′-CTGTGGGTCTCAGGGAGATCA-3′ were generated by Primer Bank, ID# 13128950a1 [63], [64]. Primers used to measure IFN-β were published previously [65]. Gene expression was normalized using the ΔΔC_t_ method, where the amount of target, normalized to an endogenous reference and relative to a calibrator, is given by 2^-ΔΔCt^, where C_t_ is the cycle number of the detection threshold.

### Statistics

For ELISA and qRT-PCR, the mean percentage of gene expression relative to control is reported, with error bars representing the S.E.M. for at least three independent experiments. For endocytosis experiments, the mean association or endocytosis index relative to control is reported, with error bars representing the S.E.M. for three independent experiments. Statistical significance was determined by Mann-Whitney U analysis.

## Supporting Information

Figure S1 shRNA targeting integrin α3 specifically reduces the expression of integrin α3. A) U937 cells were stably transduced with integrin α3-targeting shRNA (Itgα3 shRNA) or non-targeting shRNA (Ctrl. shRNA) and analyzed by qRT-PCR. All values are normalized to β-actin. Values represent mean integrin α3 expression relative to cells transduced with control shRNA and S.E.M. of three independent experiments. * p = 0.037 B) U937 cells stably transduced with integrin α3-targeting (Itgα3 shRNA) or non-targeting shRNA (Ctrl. shRNA) and analyzed by qRT-PCR. All values are normalized to β-actin. Values represent mean expression relative to cells transduced with control shRNA and S.E.M. of three independent experiments.
(3.68 MB TIF)

Figure S2 Monensin reduces expression of IL-6 mRNA in response to Pam3CSK4. U937 cells were treated with 1 µM monensin (Mon.) or control (Ctrl.), and stimulated with 100 ng/ml of Pam3CSK4 for 6 hours under serum-free conditions. IL-6 expression was analyzed by qRT-PCR and normalized to β-actin. Values represent mean transcription of IL-6 relative to control cells and S.E.M. of three independent experiments. * p = 0.037.
(1.72 MB TIF)

Figure S3 shRNA targeting TLR2 specifically reduces the expression of TLR2. A) U937 cells stably transduced with TLR2-targeting shRNA (TLR2 shRNA) or non-targeting (Ctrl. shRNA) constructs were analyzed by qRT-PCR. All values are normalized to β-actin. Values represent mean TLR2 expression relative to cells transduced with control shRNA and S.E.M. of three independent experiments. * p = 0.037 B) U937 cells stably transduced with TLR2-targeting (TLR2 shRNA) or non-targeting shRNA (Ctrl. shRNA) and analyzed by qRT-PCR. All values are normalized to β-actin. Values represent mean TLR expression relative to cells transduced with control shRNA and S.E.M. of three independent experiments.
(3.83 MB TIF)

Figure S4 Monensin reduces expression of IL-6 mRNA in response to B. burgdorferi. U937 cells were treated with 1 µM monensin (Mon.) or control (Ctrl.), and stimulated with B. burgdorferi at MOI 10 for 6 hours under serum-free conditions. IL-6 expression was analyzed by qRT-PCR and normalized to β-actin. Values represent mean transcription of IL-6 relative to control cells and S.E.M. of three independent experiments. * p = 0.037.
(1.72 MB TIF)

Figure S5 TNF-α secretion requires endosomal maturation to a lesser degree than IL-6 secretion. A) U937 cells were treated with 100 ng/ml concanamycin A (Conc.), 1 µM monensin (Mon.), or control (Ctrl.) and stimulated with 100 ng/ml Pam3CSK4 for 6 hours under serum-free conditions. Values represent mean secretion of TNF-α relative to control cells and S.E.M. of three independent experiments. Control cells secreted a mean of 860 pg/ml, concanamycin A-treated cells secreted a mean of 928 pg/ml, and monensin-treated cells secreted a mean of 567 pg/ml. * p = 0.037 B) U937 cells were treated with 1 µM monensin (Mon.) or control (Ctrl.), and stimulated with 100 ng/ml of Pam3CSK4 for 6 hours under serum-free conditions. TNF-α expression was analyzed by qRT-PCR and normalized to β-actin. Values represent mean transcription of TNF-α relative to control cells and S.E.M. of three independent experiments.
(3.45 MB TIF)
