# Supplementary figures and images for: Human Integrin α3β1 Regulates TLR2 Recognition of Lipopeptides from Endosomal Compartments
Source: PLoS One. 2010 Sep 22;5(9):e12871. doi: 10.1371/journal.pone.0012871 (PMC2943923; doi:10.1371/journal.pone.0012871)

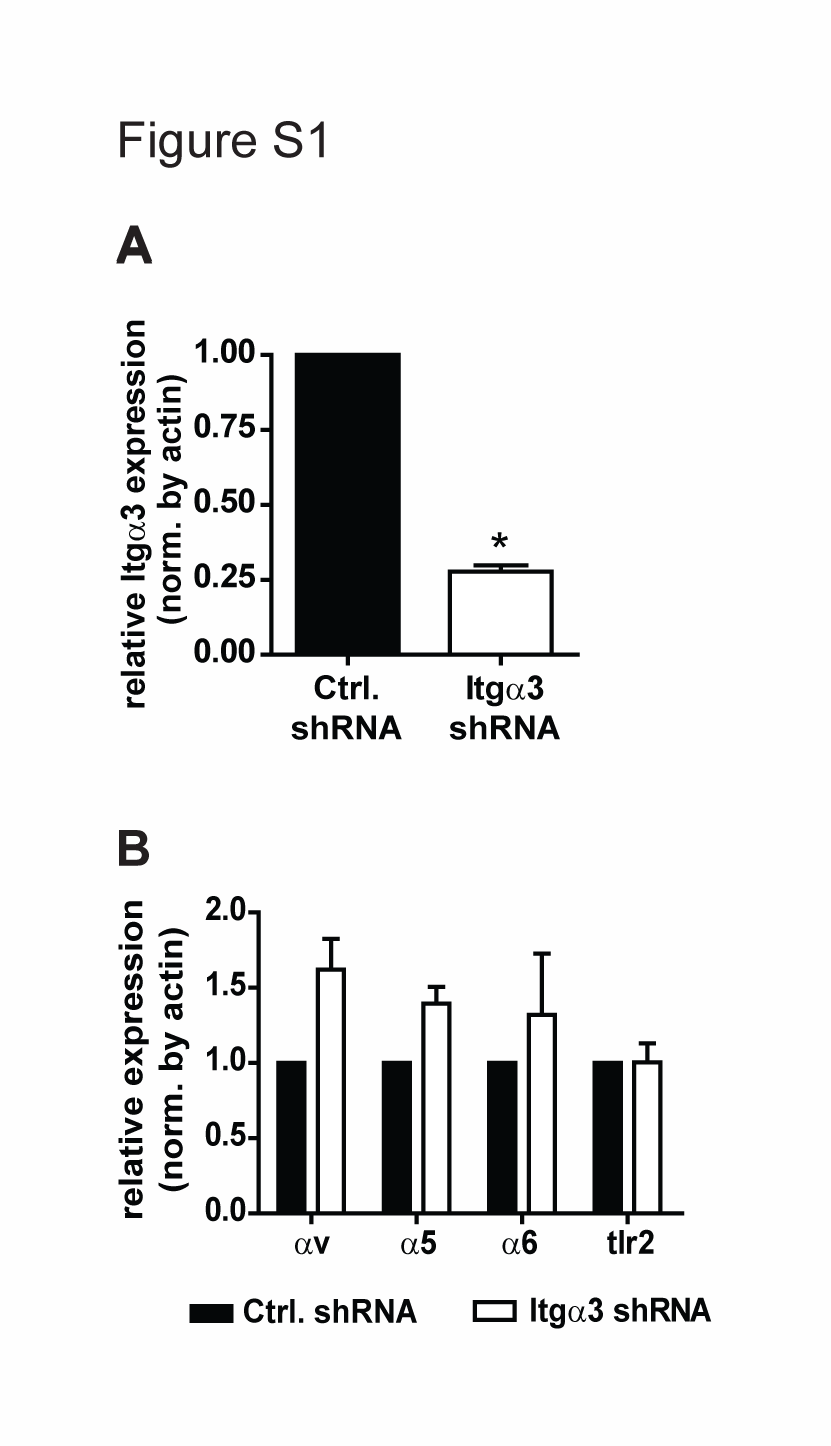

Supplement: Figure S1 — shRNA targeting integrin α3 specifically reduces the expression of integrin α3. A) U937 cells were stably transduced with integrin α3-targeting shRNA (Itgα3 shRNA) or non-targeting shRNA (Ctrl. shRNA) and analyzed by qRT-PCR. All values are normalized to β-actin. Values represent mean integrin α3 expression relative to cells transduced with control shRNA and S.E.M. of three independent experiments. * p = 0.037 B) U937 cells stably transduced with integrin α3-targeting (Itgα3 shRNA) or non-targeting shRNA (Ctrl. shRNA) and analyzed by qRT-PCR. All values are normalized to β-actin. Values represent mean expression relative to cells transduced with control shRNA and S.E.M. of three independent experiments. (3.68 MB TIF) [file pone.0012871.s001.tif]

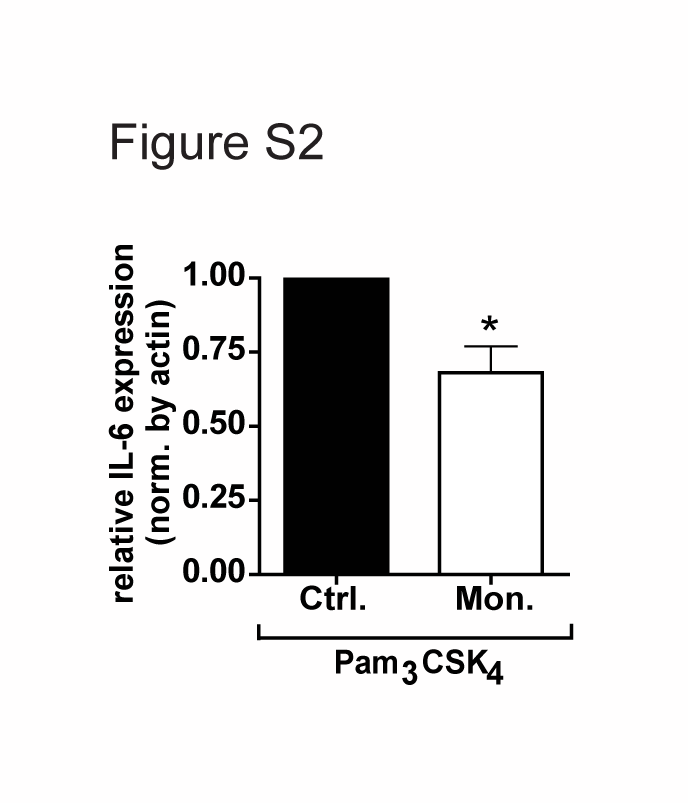

Supplement: Figure S2 — Monensin reduces expression of IL-6 mRNA in response to Pam3CSK4. U937 cells were treated with 1 µM monensin (Mon.) or control (Ctrl.), and stimulated with 100 ng/ml of Pam3CSK4 for 6 hours under serum-free conditions. IL-6 expression was analyzed by qRT-PCR and normalized to β-actin. Values represent mean transcription of IL-6 relative to control cells and S.E.M. of three independent experiments. * p = 0.037. (1.72 MB TIF) [file pone.0012871.s002.tif]

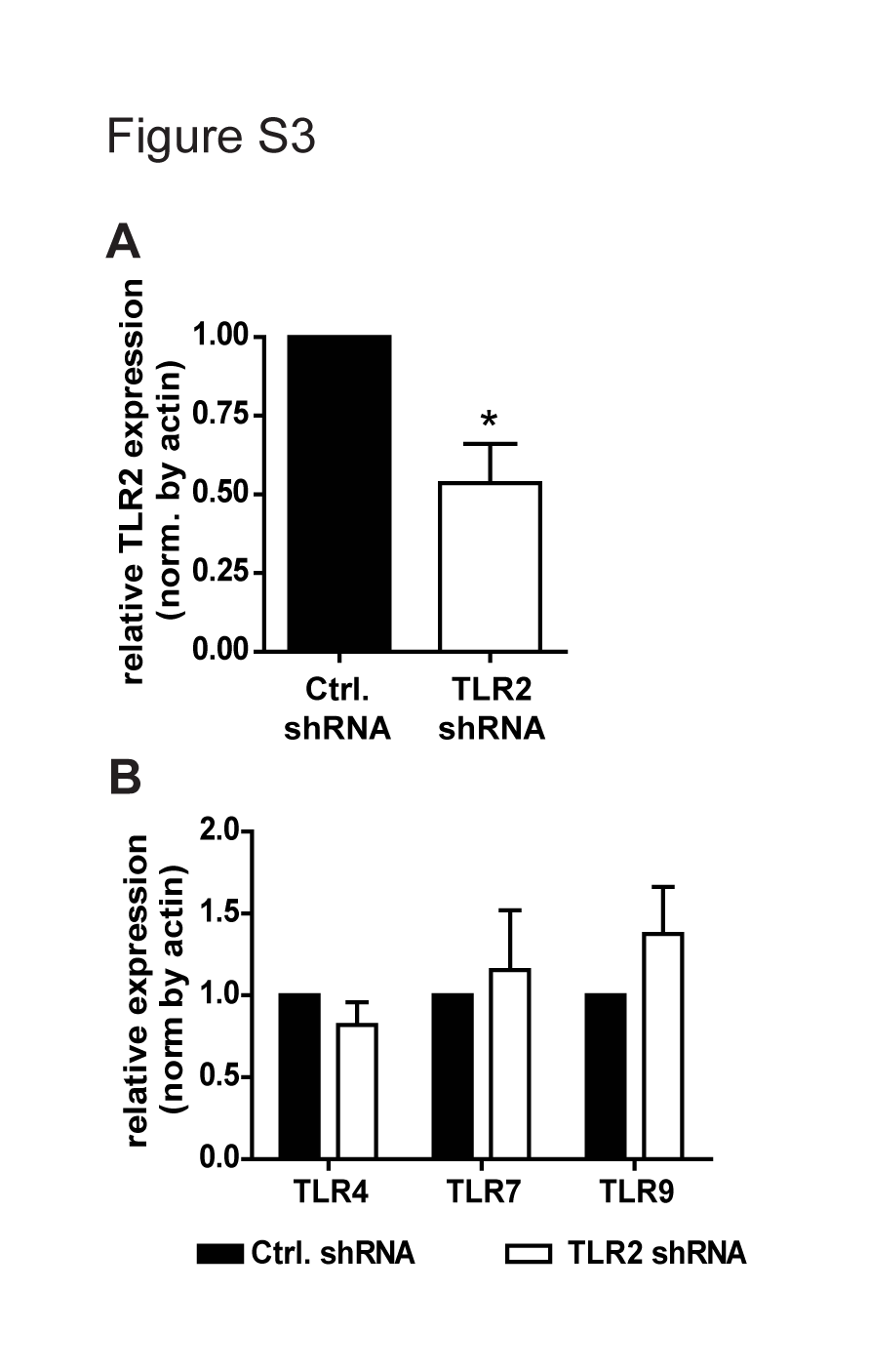

Supplement: Figure S3 — shRNA targeting TLR2 specifically reduces the expression of TLR2. A) U937 cells stably transduced with TLR2-targeting shRNA (TLR2 shRNA) or non-targeting (Ctrl. shRNA) constructs were analyzed by qRT-PCR. All values are normalized to β-actin. Values represent mean TLR2 expression relative to cells transduced with control shRNA and S.E.M. of three independent experiments. * p = 0.037 B) U937 cells stably transduced with TLR2-targeting (TLR2 shRNA) or non-targeting shRNA (Ctrl. shRNA) and analyzed by qRT-PCR. All values are normalized to β-actin. Values represent mean TLR expression relative to cells transduced with control shRNA and S.E.M. of three independent experiments. (3.83 MB TIF) [file pone.0012871.s003.tif]

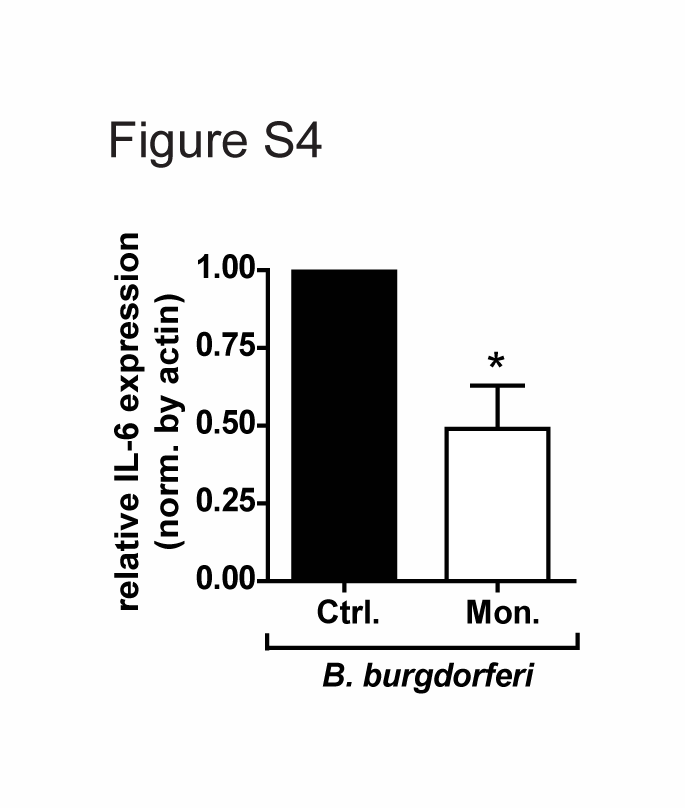

Supplement: Figure S4 — Monensin reduces expression of IL-6 mRNA in response to B. burgdorferi. U937 cells were treated with 1 µM monensin (Mon.) or control (Ctrl.), and stimulated with B. burgdorferi at MOI 10 for 6 hours under serum-free conditions. IL-6 expression was analyzed by qRT-PCR and normalized to β-actin. Values represent mean transcription of IL-6 relative to control cells and S.E.M. of three independent experiments. * p = 0.037. (1.72 MB TIF) [file pone.0012871.s004.tif]

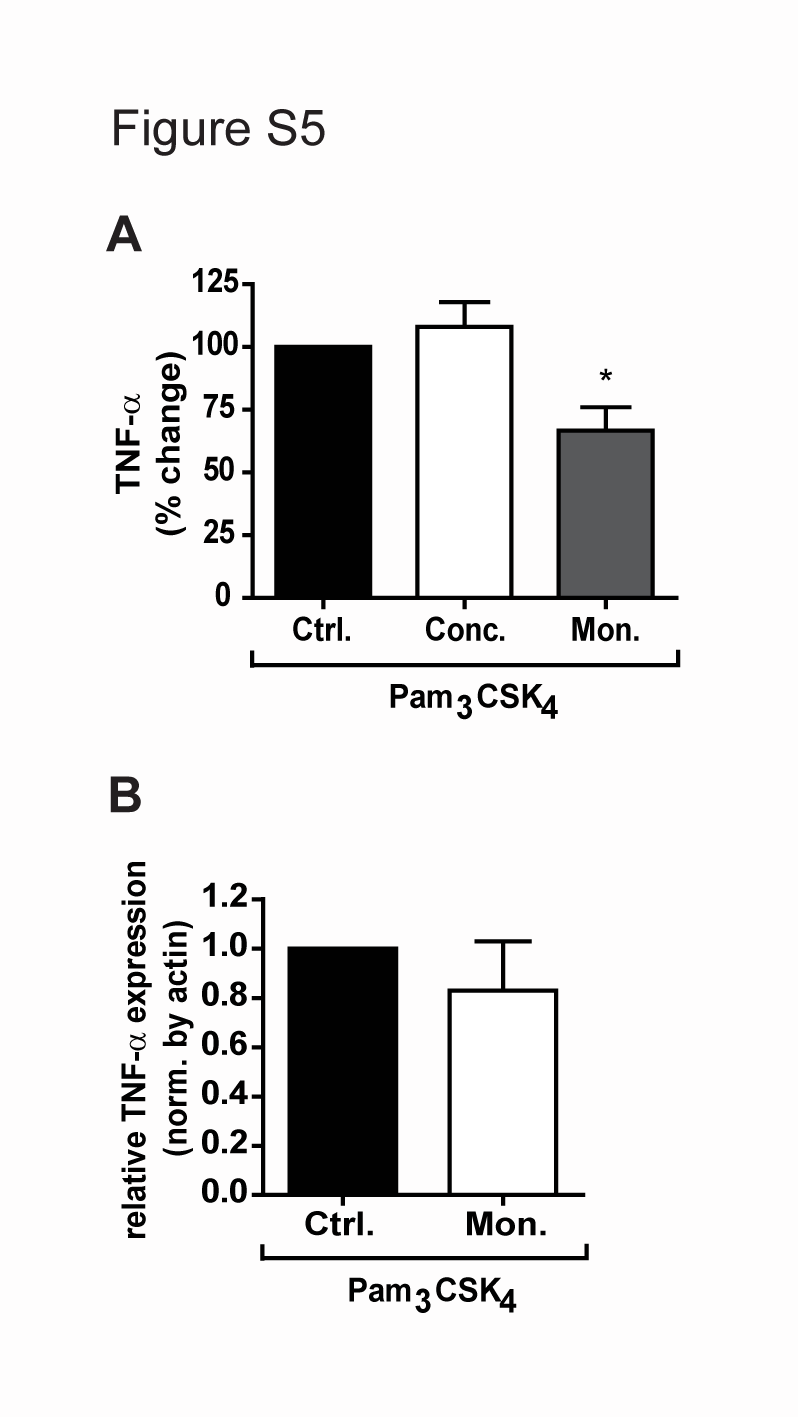

Supplement: Figure S5 — TNF-α secretion requires endosomal maturation to a lesser degree than IL-6 secretion. A) U937 cells were treated with 100 ng/ml concanamycin A (Conc.), 1 µM monensin (Mon.), or control (Ctrl.) and stimulated with 100 ng/ml Pam3CSK4 for 6 hours under serum-free conditions. Values represent mean secretion of TNF-α relative to control cells and S.E.M. of three independent experiments. Control cells secreted a mean of 860 pg/ml, concanamycin A-treated cells secreted a mean of 928 pg/ml, and monensin-treated cells secreted a mean of 567 pg/ml. * p = 0.037 B) U937 cells were treated with 1 µM monensin (Mon.) or control (Ctrl.), and stimulated with 100 ng/ml of Pam3CSK4 for 6 hours under serum-free conditions. TNF-α expression was analyzed by qRT-PCR and normalized to β-actin. Values represent mean transcription of TNF-α relative to control cells and S.E.M. of three independent experiments. (3.45 MB TIF) [file pone.0012871.s005.tif]
